# Prevalence, patterns and associated behavioural risk factors of multimorbidity in rural India: Cross-sectional analysis from the Andhra Pradesh Children and Parents Study (APCAPS)

**DOI:** 10.1371/journal.pgph.0006694

**Published:** 2026-07-30

**Authors:** Wenbo Song, Judith Lieber, Hirotomo Yamanashi, Mika Matsuzaki, Alex Lewin, Joel Gittelsohn, Matthew Hazell, Santosh Kumar Banjara, Hemant Mahajan, Santhi Bhogadi, Srivalli Addanki, Om Kurmi, Gowri Iyer, Sureshkumar Kamalakannan, Shilpa Sadanand, Bharati Kulkarni, Sanjay Kinra, Poppy A. C. Mallinson

**Affiliations:** 1 Department of Non-communicable Disease Epidemiology, London School of Hygiene & Tropical Medicine, London, United Kingdom; 2 School of Tropical Medicine and Global Health, Nagasaki University, Nagasaki, Japan; 3 Department of Methodology, London School of Economics & Political Science, London, United Kingdom; 4 Department of General Medicine, Nagasaki University, Nagasaki, Japan; 5 Department of International Health, Johns Hopkins University, Baltimore, Maryland, United States of America; 6 Indian Council of Medical Research - National Institute of Nutrition, Hyderabad, India; 7 Indian Institute of Public Health, Bengaluru, India; 8 Centre for Healthcare and Communities, Coventry University, Coventry, United Kingdom; 9 School of Communities and Education, Northumbria University, Newcastle Upon Tyne, United Kingdom; 10 Indian Council of Medical Research, New Delhi, India; PLOS: Public Library of Science, UNITED STATES OF AMERICA

## Abstract

The prevalence of multimorbidity is projected to increase substantially in India. Previous studies have focused on urban populations and older adults, who do not represent the majority of those at risk. We aimed to estimate the prevalence and investigate patterns of multimorbidity in a rural population in India and examine their associations with four common behavioural risk factors (BRFs): daily drinking, tobacco consumption, physical inactivity, and poor sleep. This cross-sectional analysis used data of all adult participants (aged ≥18 years, mean age of 35.5 years) from the third follow-up of Andhra Pradesh Children and Parents Study (APCAPS), 2010–12. We used binary logistic regression to examine the associations between BRFs, ascertained through validated questionnaires, and the prevalence of multimorbidity, defined as having two or more of 13 common chronic conditions. We used latent class analysis (LCA) to investigate common patterns of multimorbidity, followed by multinomial logistic regression to examine their associations with BRFs (categorised as binary), adjusted for age, sex, and household socioeconomic status. Of 5332 adult participants (mean age 35.5 years, standard deviation 13.8 years) included in the analysis, 14.2% had multimorbidity, the prevalence of which increased steeply with age. Current tobacco consumption (adjusted odds ratio (aOR) = 1.23 [95% confidence interval (CI): 1.01-1.50]), physical inactivity (aOR: 1.66 [95% CI: 1.38-1.99]), and poor sleep (aOR: 1.86 [95% CI: 1.35-2.57]) were associated with higher odds of having multimorbidity. We found one multimorbidity pattern of “anaemia and hypertension” (n = 220, 4.1%). Physical inactivity (aOR: 2.07 [95% CI: 1.52-2.82]) was associated with higher odds of having this multimorbidity pattern, with no associations found for the other BRFs. The BRFs identified, notably physical activity, could present important opportunities for intervention to reduce the burden of multimorbidity in India. Longitudinal studies are needed to confirm these relationships.

## Introduction

Rural India has a population of 900 million, accounting for nearly seventy percent of the total Indian population. Following improved longevity and rapid socioeconomic and behavioural transition [1], it is projected that an increasing proportion of rural Indian adults will experience multimorbidity [2,3], defined as the co-existence of two or more chronic conditions. The prevalence of multimorbidity in rural India is estimated to be as high as 54.8% in Punjab state [4], which is at a more advanced stage of economic transition. Multimorbidity has been identified as a substantial threat to fragile rural health infrastructures in India, potentially exacerbating health inequalities experienced by vulnerable households. Concern over the growing burden of multimorbidity has prompted calls for research into its specific determinants and manifestations in the rural Indian population [5]. However, existing research is primarily focused on healthcare utilisation, out-of-pocket expenditure, and quality of life of multimorbidity, instead of modifiable risk factors for multimorbidity [6].

Behavioural risk factors (BRFs) are likely to be important targets in the prevention and management of multimorbidity, which is often comprised of chronic conditions that share common behavioural causes [7]. Evidence from global studies suggests that certain BRFs, such as daily drinking, tobacco consumption, physical inactivity, and poor sleep, are linked to a higher prevalence of multimorbidity and specific multimorbidity patterns [8–11]. Although some Indian studies have investigated BRFs for multimorbidity, these were limited to the investigation of alcohol and tobacco consumption, with limited examination on whether these associations were independent of other BRFs [5]. Compared to those who dwell in affluent areas of high-income countries (HICs) [12], multimorbidity is estimated to occur 10–15 years earlier in those who dwell in rural areas of low- and middle-income countries (LMICs). It is also widely reported that Indian populations experience common chronic conditions (such as diabetes, hypertension, heart disease) around 10 years younger than American and European populations [13]. Despite this, previous studies in India have focused on urban populations and older adults, who do not represent the majority of individuals at risk of multimorbidity in India [3]. Furthermore, despite evidence from global studies that specific patterns (combined clusters of chronic conditions) of multimorbidity may be associated with particular BRFs [7,8], this has not been investigated in the Indian context [14]. This has hindered understanding of the causal links between specific clusters of conditions relevant to the Indian context, including their underlying BRFs, which may guide targets for interventions to prevent multimorbidity that are appropriate for this setting [15].

We used cross-sectional data from the third wave of the Andhra Pradesh Children and Parents Study (APCAPS), a rural population-based cohort including primarily young and middle-aged adults with detailed information on a range of BRFs, to examine the burden of multimorbidity and determine its BRFs in rural India [16]. Our objectives were to [1] estimate the prevalence of multimorbidity and investigate the multimorbidity patterns and [2] examine the association of BRFs with overall multimorbidity and specific multimorbidity patterns.

## Materials and methods

### Study population

The present cross-sectional analysis used data from the third wave of the APCAPS cohort (see S1 File), a prospective study involving 29 rural towns and villages near Hyderabad city, Telangana state, India [16]. Data for this wave were collected in 2010–12 from participants of the earlier established Hyderabad Nutrition Trial (HNT) and their siblings and parents (n = 6,944; around 70% of potentially eligible participants). We included the data on all adult participants aged ≥18 years.

### Ethics statement

The APCAPS study received ethical approval from the National Institute of Nutrition (Hyderabad, India), the Indian Council of Medical Research (New Delhi, India), and the London School of Hygiene and Tropical Medicine (London, United Kingdom). Prior to data collection, approval had been sought from the village heads and their panchayats in each of the 29 villages. Written informed consent (or witnessed thumbprint if illiterate) was obtained from all participants [16].

This analysis was reviewed by the executive team of the Andhra Pradesh Children and Parents Study [17] and the Ethics Board of the London School of Economics and Political Science (Approval Number No.44760). We accessed the data for research purposes on July 21, 2020.

### Measures

We examined 13 common chronic conditions, based on the availability of either or both self-reported history and symptom-based screening. The full list of definitions and measurement instruments for each is presented in **S2 File**. These, coded as binary variables, included: anaemia, asthma (asthma, asthmatic bronchitis, or allergic bronchitis), chronic kidney disease, diabetes mellitus, heart disease, or hypertension, chronic obstructive pulmonary disease (chronic bronchitis or emphysema), mental disorder (depression or anxiety), peptic ulcer disease, sarcopenia, stroke, thyroid problem, and tuberculosis. We defined multimorbidity as the co-existence of two or more chronic conditions in a single individual, which is the most common definition of multimorbidity used in epidemiological studies [18], and we defined multimorbidity patterns as the specific probabilistic co-existence of chronic conditions across individuals.

We analysed 4 BRFs with binary categories, which were identified as risk factors for multimorbidity based on recent systematic reviews [8,9]: daily drinking (weekly vs monthly/daily), tobacco consumption (never vs current/former), physical inactivity (sedentary vs active, based on a locally validated physical activity questionnaire), and poor sleep (6–10 hours per day vs < 6 or ≥ 10 hours per day) [19–22]. BRF information was collected through self-reported questionnaires (available at https://www.lshtm.ac.uk/APCAPS). We did not examine diet, another potential BRF for multimorbidity, because diet is highly complex and multifaceted and it was not deemed feasible to summarise dietary information into a meaningful binary indicator for dietary quality that could be analysed in a consistent manner alongside the other BRFs. We considered the following sociodemographic factors as potential confounders, based on their relevance to multimorbidity in existing studies: age, sex, education, and household socioeconomic status (SES) defined by the Indian National Family Health Survey (NFHS)’s definition of the standard of living index [8,23–25].

### Statistical analyses

Descriptive statistics were used to summarise the characteristics of the study population. The count and proportion of chronic conditions were reported, stratified by age group (18–29 years, 30–44 years, 45–59 years, 60 + years) and sex (female and male). Unadjusted and adjusted binary logistic regression was used to examine the associations between BRFs and the prevalence of multimorbidity. In the adjusted model, we examined the associations between all four BRFs and the prevalence of multimorbidity, controlling for the other BRFs, age, sex, education, and household SES.

To investigate multimorbidity patterns, we used Latent Class Analysis (LCA), an unsupervised machine learning approach for describing the relationships among categorical variables. LCA produces a probabilistic clustering of individuals into latent classes. Each latent class represents a particular pattern of chronic conditions. LCA simultaneously estimates the latent classes and the probability of class membership for every individual. Thus, LCA enabled us to identify conditions that were highly represented in the dataset and correlated with each other, from which we selected the most common conditions within each latent class to define multimorbidity patterns for subsequent regression analyses based on probabilistic statistics of each condition. A detailed explanation of the methodology is presented in **S3 File**. Participants without multimorbidity or those having no disease were included in LCA to minimise the overfitting of cluster analysis. Model selection was determined on the optimal Bayesian information criterion (BIC) values, with lower BIC values indicating a better-fitting model. The subsequent analysis examined the associations between BRFs and the identified multimorbidity pattern using multinomial logistic regression (comparing between no pattern, single condition from the pattern, and the identified pattern). In the adjusted model, we examined the associations between all four BRFs and the multimorbidity pattern, controlling for age, sex, education, household SES, and multimorbidity status (defined as no identified pattern).

All data analyses were performed in the R version 4.1.2, with functions implemented in the R packages: foreign, MASS, nnet, poLCA, tidyverse, tidymodels, scatterplot3d, stats, and transplantr.

## Results

Of the 6,944 cohort participants recruited to APCAPS between 2010 and 2012, we excluded those under 18 years of age (n = 646) and those with missing values for any disease outcomes or BRFs (n = 966), resulting in 5332 participants included in the analyses (**Table 1**). The participants were 45.7% female and relatively young, with a median age of 31 years and a mean age of 35.5 years (interquartile range 23–47 years; standard deviation = 13.8). The age distribution is reported in **S4 File**. The majority belonged to the low education group and had household SES in the highest tertile nationally. Despite only 2.1% of the sample consuming alcohol daily, two-thirds were physically inactive, one-quarter consumed tobacco, and one-eighth slept poorly.

**Table 1:** The characteristics of the adult participants (n = 5332).

Characteristics	*n* (%)
Age (years)	
18-29	2545 (47.7)
30–44	990 (18.6)
45–59	1516 (28.4)
>=60	281 (5.3)
Sex	
Female	2437 (45.7)
Male	2895 (54.3)
Education	
Illiterate or no formal education	2590 (48.6)
Up to high school	2295 (43.0)
University	447 (8.4)
Household Socioeconomic Status (SES)	
High (25+)	3494 (65.5)
Medium (15 –24)	1619 (30.4)
Low (0–14)	219 (4.1)
Daily drinking	
No	5222 (97.9)
Yes	110 (2.1)
Tobacco Consumption	
Never	3948 (74.0)
Current or former	1384 (26.0)
Physical Inactivity	
Active	1748 (32.8)
Sedentary	3584 (67.2)
Poor Sleep	
6-10 hours / day	5012 (94.0)
<6 or ≥10 hours / day	320 (6.0)
Chronic Conditions	
Anaemia	1316 (24.7)
Asthma (including allergic bronchitis)	54 (1.0)
Chronic kidney disease	79 (1.5)
Diabetes mellitus	243 (4.6)
Heart disease	7.9 (1.5)
Hypertension	1061 (19.9)
Chronic obstructive pulmonary disease (including chronic bronchitis and emphysema)	10 (0.2)
Mental disorder (depression, anxiety)	81 (1.5)
Peptic ulcer disease	57 (1.1)
Sarcopenia	604 (11.3)
Stroke	28 (0.5)
Thyroid problem	30 (0.6)
Tuberculosis	20 (0.4)
Multimorbidity	
Yes	755 (14.2)
No	4577 (85.8)

**Fig 1:**
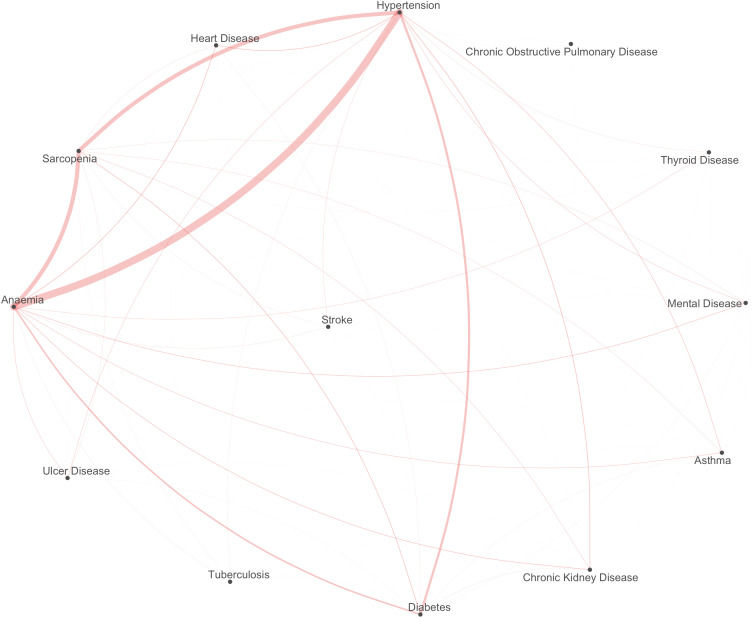
Feature co-occurrence matrix (n = 5332).

The four most prevalent chronic conditions were anaemia, hypertension, sarcopenia, and diabetes, and these four conditions frequently co-occurred (**Fig 1**). Overall, 14.2% of adult participants in APCAPS had multimorbidity. The prevalence of multimorbidity increased with age, from 2.8% among those aged 18–29 years to 49.5% among those aged ≥ 60 years (**S5 File**). Notably, multimorbidity was present in a substantial proportion of middle-aged adults (14.9% aged 30–44 and 26.1% aged 45–59). Among the younger age group (18–44 years), the proportion of female participants with multimorbidity was higher than that of male participants, whereas in the older age groups (≥ 45 years), the proportion was lower for females compared to males.

### Associations between BRFs and multimorbidity prevalence

The distributions of BRFs for participants with and without multimorbidity are reported in **S6 File**. Compared to participants without multimorbidity, those with multimorbidity had a higher prevalence of daily drinking, tobacco consumption and poor sleep but lower rates of physical inactivity. The univariable binary logistic analyses indicated that daily drinking (OR = 1.81 [95% CI: 1.15, 2.85]), tobacco consumption (OR = 2.75 [95% CI: 2.35, 3.23]), and poor sleep (OR = 1.70 [95% CI: 1.28, 2.25]) were associated with a higher prevalence of multimorbidity. In the multivariable logistic regression analysis, we found that tobacco consumption (aOR = 1.23 [95% CI: 1.01, 1.50]), physical inactivity (aOR = 1.66 [95% CI: 1.38, 1.99]) and poor sleep (aOR = 1.86 [95% CI: 1.35, 2.57]) were each positively associated with multimorbidity (**Table 2**).

**Table 2:** Binary logistic regression analysis of BRFs for multimorbidity prevalence (n = 5332).

Characteristics	*Univariable analysis*			*Multivariable analysis*	
	*OR (95% CI)*	*P*		*aOR (95% CI)*	*P*
Daily drinking					
No (N = 5222)	1.0			1.0	
Yes (N = 110)	**1.81 (1.15, 2.85)**	**<0.0001**		0.94 (0.57, 1.54)	0.80
Tobacco consumption					
No (N = 3948)	1.0			1.0	
Current or former (N = 1384)	**2.75 (2.35, 3.23)**	**<0.0001**		**1.23 (1.01, 1.50)**	**0.04**
Physical inactivity					
Active (N = 1748)	1.0			1.0	
Sedentary (N = 3584)	0.91 (0.77, 1.07)	0.26		**1.66 (1.38, 1.99)**	**<0.0001**
Poor sleep					
6-10 hours / day (N = 4662)	1.0			1.0	
<6 or ≥10 hours / day (N = 670)	**1.70 (1.28, 2.25)**	**0.0002**		**1.86 (1.35, 2.57)**	**0.0002**
Age (years)	.			**1.09 (1.08, 1.10)**	**<0.0001**
Sex					
Male	.			1.0	
Female	.			**1.44 (1.18, 1.76)**	**0.0004**
Education					
University	.			1.0	
Up to high school	.			1.52 (0.80, 2.88)	0.20
Illiterate or no formal education	.			1.42 (0.77, 2.62)	0.27
Household Socioeconomic Status (SES)					
High (25+)	.			1.0	
Medium (15–24)	.			0.70 (0.46, 1.05)	0.08
Low (0–14)	.			0.87 (0.72, 1.04)	0.13

* OR: Odds ratio; aOR: adjusted Odds ratio; CI: Confidence interval; Multivariable model adjusted for age, sex, education, and household socioeconomic status

### Pattern of multimorbidity

**Fig 2:**
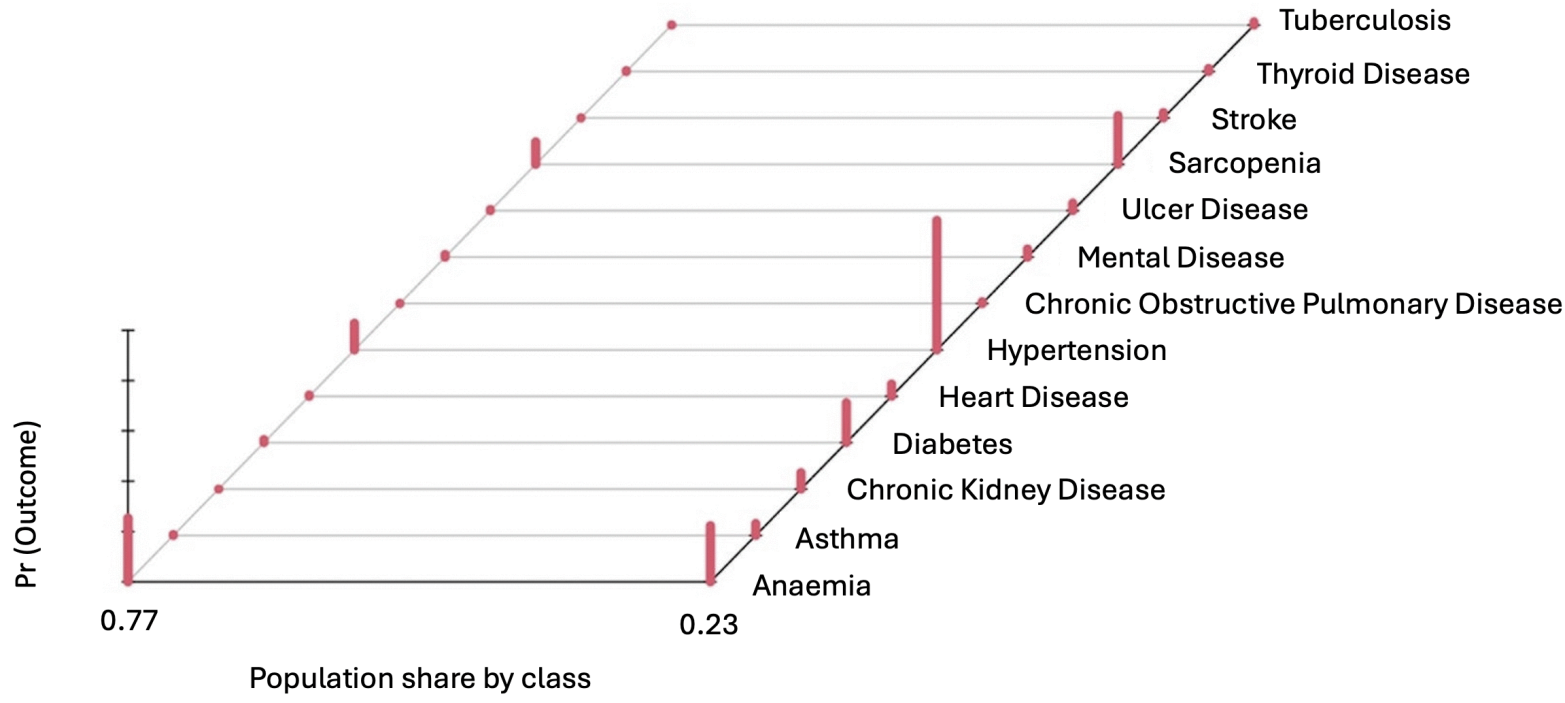
Estimation of the two-class latent class model (n = 5332).

The pattern of multimorbidity among prevalent conditions was explored using LCA (**S7 File**). We selected a model with two clusters based on the lowest BIC value and found one multimorbidity pattern of “anaemia (conditional probability (CP) = 0.22) and hypertension (CP = 0.51)” from the same cluster, as shown in **Fig 2**. 622 (11.7%) participants were assigned to the first class that contains this pattern, and 4710 (88.3%) participants were assigned to a cluster in which no chronic conditions had conditional probability ≥ 0.2. 220 (4.1%) participants had both anaemia and hypertension. Other prevalent conditions in this cluster were sarcopenia (CP = 0.19) and diabetes (CP = 0.15).

### Associations between BRFs and multimorbidity pattern

The distributions of BRFs for participants with and without the multimorbidity pattern of “anaemia and hypertension” are shown in S8 File. Daily drinking was most common in the hypertension-only group. Tobacco consumption was common among people with hypertension, as well as people with the multimorbidity pattern of “anaemia and hypertension”. Physical inactivity and poor sleep were relatively consistent across all groups. In univariable analyses, we only found clear evidence for an association between tobacco consumption and the pattern of “Anaemia and Hypertension” (OR = 1.93 [95% CI: 1.45, 2.56]). In multivariable multinomial analysis, we only found clear evidence for an association with physical inactivity (aOR = 2.07 [95% CI: 1.52, 2.82]) (**Table 3**).

**Table 3:** Multinomial logistic regression analysis of BRFs for multimorbidity pattern (n = 5332).

Behavioural risk factors (BRFs)	No anaemia or hypertension (n = 3175)	Anaemia only (n = 1096) *OR (95% CI)*	Hypertension only (n = 841) *OR (95% CI)*	Pattern “Anaemia and Hypertension” (n = 220) *OR (95% CI)*
Daily drinking (N = 110)				
Univariable	1.0	0.39 (0.17, 0.85)	**3.80 (2.56, 5.64)**	0.27 (0.04, 1.99)
Multivariable	1.0	1.10 (0.48, 2.50)	**2.22 (1.44, 3.43)**	0.23 (0.03, 1.70)
Tobacco consumption (N = 1384)				
Univariable	1.0	0.60 (0.50, 0.72)	**2.27 (1.94, 2.66)**	**1.93 (1.45, 2.56)**
Multivariable	1.0	1.09 (0.87, 1.35)	1.09 (0.89, 1.32)	1.24 (0.88, 1.73)
Physical inactivity (N = 3584)				
Univariable	1.0	0.92 (0.80, 1.07)	0.92 (0.78, 1.08)	0.95 (0.71, 1.27)
Multivariable	1.0	1.14 (0.96, 1.35)	**1.40 (1.16, 1.67)**	**2.07 (1.52, 2.82)**
Poor sleep (N = 320)				
Univariable	1.0	**1.61 (1.23, 2.11)**	1.33 (0.97, 1.81)	0.78 (0.39, 1.54)
Multivariable	1.0	**1.49 (1.11, 2.00)**	**1.46 (1.04, 2.04)**	0.71 (0.35, 1.45)

* Multivariable model adjusted for age, sex, education, and household socioeconomic status

## Discussion

In this sample of adults living in rural India, multimorbidity increased significantly with age, although it was notably prevalent even in mid-adulthood (16% of women and 12% of men aged 30–45 years). We found that tobacco consumption, physical inactivity, and poor sleep were associated with a higher prevalence of multimorbidity. We also identified a multimorbidity pattern of “anaemia and hypertension”, which was associated with physical inactivity but not the other BRFs examined.

Our findings are aligned with a large body of global and regional evidence that shows a significantly higher prevalence of multimorbidity in older adults than in younger adults [14,26] and in females than in males [27]. We found the prevalence rate of multimorbidity was notable in middle-aged adults (14.9 percent in ages 30–44) and rose steeply to 49.5 percent in those aged 60 and over. It suggests that the older rural population may bear a higher chronic disease burden. The overall estimated prevalence of multimorbidity among young and middle-aged cohort participants (14.2 percent) is below the national pooled estimate of 20.0 percent in the latest systematic review [3]. Within the younger group, the high prevalence of multimorbidity is partly accounted for by the high prevalence of anaemia, which is higher among young females than males. Although daily drinking is widely known to be a risk factor for many chronic conditions, such as ischemic heart disease and stroke in HICs, our finding of no association between alcohol and multimorbidity is in line with recent synthesised evidence from India (pooled OR = 1.16 [95% CI: 0.92 to 1.46]) [5]. However, our wide confidence intervals (due to low prevalence of regular alcohol use) are also consistent with findings of moderate effect sizes (OR: 1.3) in two national Indian studies (n = 72250; n = 9852) [28–30]. The true association between daily drinking and multimorbidity may vary by country or even region of India in light of the types of alcoholic beverages consumed [8], while reporting bias related to stigma could also contribute to heterogeneity in the published evidence. We found that people who consumed tobacco had 23% increased odds of multimorbidity, consistent with recent evidence in India (OR: 1.33 [95% CI: 1.16–1.52]) and the known harmful effects of smoking on multiple organ systems [5]. We found that physically inactive people had over 60 percent increased odds of multimorbidity. This association only became apparent after adjusting for sociodemographic factors, which was primarily driven by the confounding effect of age, given the higher levels of physical inactivity among younger participants. This is consistent with some evidence from other LMICs [31], although this has not been examined before in India. We found that people who reported poor sleep had 86% increased odds of multimorbidity, a novel observation in the Indian population. This has biological plausibility as poor sleep is linked to multiple chronic conditions as well as being a marker of stress, which is thought to contribute to the risk of multimorbidity [9]. Although we were unable to stratify by long and short sleep duration due to low numbers, a recent systematic review has suggested that the pooled odds ratio for the association with multimorbidity was strong for both short (pooled ORs = 1.48) and long sleep duration (pooled ORs = 1.21) [9,32].

The multimorbidity pattern of “anaemia and hypertension” that we identified is widely reported globally [33], as well as in previous studies based on national Indian surveys among all adults (prevalence = 2.2 percent) and among women aged 15–49 years (prevalence = 0.63 percent) [34,35]. It presents a public health concern to see the multimorbidity pattern of anaemia and hypertension among younger adults in this rural Indian context, requiring further research into its causes to inform context-appropriate preventive interventions [36]. Although tobacco consumption was found to be associated with the prevalence of multimorbidity, the association is weaker with this pattern. This further adds to the existing inconsistent evidence base about this association in similar contexts [37]. Our study found that the odds of experiencing this multimorbidity pattern were more than doubled among those who were physically inactive. Although physical inactivity is a known determinant of hypertension and may contribute to anaemia [38–40], this cross-sectional association should be interpreted cautiously, as physical inactivity can also be a consequence of anaemia, as people with anaemia are more likely to be tired and short of breath [41]. Our study did not find other multimorbidity patterns that have been recognised globally, such as “cardio-metabolic” [42], or specific patterns identified in India, such as “diabetes and hypertension” and “arthritis and hypertension” [3]. This may be due to the relatively young age of our study sample, which meant that ageing-related chronic conditions were less common. Also, this may result from the specific methodology we used to define a multimorbidity pattern, as some conditions, such as sarcopenia and diabetes, were represented but not prevalent in the multimorbidity cluster we identified. We note that the above comparisons between our findings and other literature on BRFs for multimorbidity should be interpreted with caution due to the wide variation in how different studies have defined multimorbidity, its specific patterns, and how these are measured [43].

To the best of our knowledge, this is the first study to examine several common BRFs associated with both prevalence and specific patterns of multimorbidity in adults living in India [3,8]. Most previous risk factor studies conducted in India have only used a simple count of chronic conditions to define prevalent multimorbidity. In contrast, our study explored the associations between BRFs and a specific multimorbidity pattern that we identified quantitatively. Moreover, we included 13 common chronic conditions across all adult age groups, in comparison to earlier Indian studies in which the median number of included chronic conditions was 9 and focused on older populations [3]. Furthermore, as many chronic conditions remain undiagnosed in India, we used validated clinical measures to classify 8 out of 13 chronic conditions, rather than relying solely on self-reported diagnoses as most previous studies have done [44]. However, our data were cross-sectional, meaning that it is not possible to establish the temporal ordering of exposure and outcome; longitudinal studies are needed to clarify this. We also relied on self-reported disease diagnoses for some conditions, such as asthma and stroke, which would have likely led to an underestimation of prevalence and patterns of multimorbidity, while our use of self-reported measures for BRFs could have biased associations in either direction. Existing medication uses and adverse event interactions can also bias associations by altering presentations of chronic conditions. Although we used the most common clustering method, latent class analysis, to identify multimorbidity patterns [45], the low prevalence of several chronic conditions limited the overall variance in co-occurrence, resulting in a single multimorbidity pattern that primarily reflects disease burden rather than distinct phenotypic subtypes. The fact that we only included 13 conditions may have also limited our ability to detect certain patterns, especially those containing rarer diseases not captured in our study. We cannot rule out residual confounding, such as due to diet, genetic factors, or inaccurately measured socioeconomic factors, which could have inflated our observed associations [24]. We excluded participants with missing data. The complete case analysis implicitly assumes that the data are missing at random, which is plausible in this scenario but unverifiable.

The recent rapid economic transition and unplanned urbanisation in India have made rural populations more vulnerable to various health risk factors, and the increased life expectancy means that they are more likely to become multimorbid and spend longer periods living with multimorbidity [46]. Our study highlights the substantial prevalence of multimorbidity, even at relatively young ages, in rural populations in India. We found that several BRFs, namely tobacco use, physical inactivity, and poor sleep, were associated with multimorbidity. Additionally, physical inactivity was associated with the multimorbidity pattern “anaemia and hypertension”. This study supports ongoing policy efforts to improve population levels of these BRFs in the Indian population, given the growing burden of multimorbidity [47]. Further research should take diet into analysis and aim to establish the causality of these associations to inform more targeted behavioural interventions to prevent and appropriately manage multimorbidity in this setting.

## Conclusion

Our study demonstrated a higher prevalence of multimorbidity among the elderly in a rural population in southeastern India. Multimorbidity was associated with tobacco consumption, physical inactivity, and poor sleep. We found a multimorbidity pattern of “anaemia and hypertension” in this cohort, and this pattern was associated with physical inactivity. It is possible that these BRFs could present important opportunities for intervention, aiming to reduce the burden of multimorbidity in the Indian population.

## Supporting information

S1 File Flowchart of the analytical sample selection for the participants of the Andhra Pradesh Children and Parents Study (2010–2012). (DOCX)

S2 File Selected chronic conditions and collection instruments from the survey in 2010–12. (DOCX)

S3 File Explanation of the methodology for latent class analysis (LCA). (DOCX)

S4 File Age distribution (n = 5332). (DOCX)

S5 File Prevalence of chronic condition by age and sex groups (n = 5332). (DOCX)

S6 File The distribution of BRFs between those with multimorbidity and those without multimorbidity (n = 5332). (DOCX)

S7 File Comparison between models of latent class analysis (LCA) (n = 5332). (DOCX)

S8 File The distribution of BRFs between those with the multimorbidity pattern and those without the multimorbidity pattern (n = 5332). (DOCX)
