# Supplementary material for: Prevalence, patterns and associated behavioural risk factors of multimorbidity in rural India: Cross-sectional analysis from the Andhra Pradesh Children and Parents Study (APCAPS)
Source: PLOS Glob Public Health. 2026 Jul 30;6(7):e0006694. doi: 10.1371/journal.pgph.0006694 (PMC13422877; doi:10.1371/journal.pgph.0006694)
Supplement: S1 File — (DOCX) [file pgph.0006694.s001.docx]

**Online** **Supplemental File 1.** Flowchart of the analytical sample selection for the participants of the Andhra Pradesh Children and Parents Study (2010-2012).

| Index children  (n = 1360)  Mothers of index children  (n = 1379),  Fathers of index children  (n = 1140),  Siblings of index children  (n = 2344)  General population not linked to the NHT  (n = 721)  Observations excluded in the analysis due to have one or more missing values  (n = 966)  Index children included in the third wave in 2010-2012  (n = 1360)  Full APCAPS sample in the third survey wave in 2021-2012  (n = 6944)  Younger than 18 years  (n = 646)  Adults, 18 year or older  (n = 6298)  Observations analysed  (n = 5332) |
| --- |
| ^*^ The Hyderabad Nutrition Trial (1987–1990) included 15 intervention and 14 control villages from the Integrated Child Development Services Scheme (stepped-wedge cluster randomised nutrition supplementation trial). APCAPS=Andhra Pradesh Children and Parents Study |
