# Supplementary material for: Prevalence, patterns and associated behavioural risk factors of multimorbidity in rural India: Cross-sectional analysis from the Andhra Pradesh Children and Parents Study (APCAPS)
Source: PLOS Glob Public Health. 2026 Jul 30;6(7):e0006694. doi: 10.1371/journal.pgph.0006694 (PMC13422877; doi:10.1371/journal.pgph.0006694)
Supplement: S2 File — (DOCX) [file pgph.0006694.s002.docx]

**Online** **Supplemental File 2.** Selected chronic conditions and collection instruments from the survey in 2010-12**.**

| **Chronic conditions** | **Self-reported history^*^** | **Extra Symptom-based Screening Instruments** |
| --- | --- | --- |
| Anaemia | . | ✓: Full blood count hemoglobin <12 for female and <13g/dL for male |
| Asthma (including allergic bronchitis) | ✓ | . |
| Chronic kidney disease | . | ✓: Estimated glomerular filtration rate < 60, calculated from serum creatinine level |
| Diabetes | ✓ | ✓: Fasting blood glucose ≥ 126 mg/dl of venous blood, assayed by enzymatic oxidation |
| Heart disease | ✓ | ✓: Rose Angina Questionnaire |
| Hypertension | ✓ | ✓: Brachial blood pressure systolic blood pressure ≥ 140 mmHg and/or diastolic blood pressure ≥ 90 mmHg |
| Chronic obstructive pulmonary disease (including chronic bronchitis and emphysema) | ✓ | ✓: Chronic obstructive pulmonary disease (COPD) assessment test |
| Mental disorder (Depression, Anxiety) | ✓ | ✓: Brief Patient Health Questionnaire |
| Peptic ulcer disease | ✓ | . |
| Sarcopenia | . | ✓: Hand grip strength < 18kg and skeletal muscle mass < 5.7 (4.61 for those aged < 40 years old) kg/m^2^ for female and < 27.5kg and skeletal muscle mass < 7.0 (6.11 for those aged < 40 years old) kg/m^2^ for male |
| Stroke | ✓ | . |
| Thyroid problem | ✓ | . |
| Tuberculosis | ✓ | . |
| ^*^ Self-reported history: the participants had diagnosis report or reported receiving medical diagnosis when attended clinics prior to the study | | |
