# Supplementary material for: Prevalence, patterns and associated behavioural risk factors of multimorbidity in rural India: Cross-sectional analysis from the Andhra Pradesh Children and Parents Study (APCAPS)
Source: PLOS Glob Public Health. 2026 Jul 30;6(7):e0006694. doi: 10.1371/journal.pgph.0006694 (PMC13422877; doi:10.1371/journal.pgph.0006694)
Supplement: S3 File — (DOCX) [file pgph.0006694.s003.docx]

**Online** **Supplemental File 3.** Explanation of the methodology for latent class analysis (LCA).

Latent class analysis (LCA) is a Range-Query (R-Q) type analysis, which focuses on both subjects (rows) and variables (columns). It uses maximum likelihood estimation to identify subgroups of cases. This method is superior to more traditional clustering procedures such as exploratory factor analysis and k-means cluster analysis, as it provides probability-based classification, a principled way to determine the number of clusters and low rates of misclassification. The cons of LCA include: (1) identified clusters may not always correspond to true subject groups, and the method assumes heterogeneous data can be explained by homogeneous subgroups, potentially leading to superfluous classes due to data nonnormality or model misspecification; (2) small sample sizes can result in poor fit indices and convergence issues. We used an LCA model designed for binary data (binomial model) in this study given that (a) multimorbidity patterns are conceptualised as discrete categories that are reflections of a single underlying cause; (b) we had a relatively large sample; and (c) we aimed to classify subjects into more than one group.
