# Supplementary figures and images for: Prevalence, patterns and associated behavioural risk factors of multimorbidity in rural India: Cross-sectional analysis from the Andhra Pradesh Children and Parents Study (APCAPS)
Source: PLOS Glob Public Health. 2026 Jul 30;6(7):e0006694. doi: 10.1371/journal.pgph.0006694 (PMC13422877; doi:10.1371/journal.pgph.0006694)

**Online** **Supplemental File 4.** Age distribution (n = 5332).


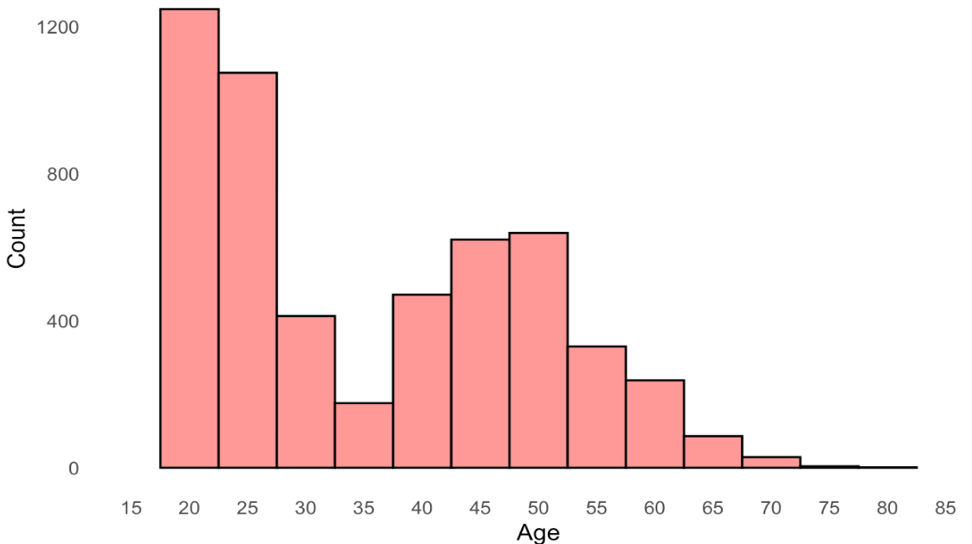

Supplement: S4 File — (DOCX) [file pgph.0006694.s004.docx]
