# Supplementary material for: Prevalence, patterns and associated behavioural risk factors of multimorbidity in rural India: Cross-sectional analysis from the Andhra Pradesh Children and Parents Study (APCAPS)
Source: PLOS Glob Public Health. 2026 Jul 30;6(7):e0006694. doi: 10.1371/journal.pgph.0006694 (PMC13422877; doi:10.1371/journal.pgph.0006694)
Supplement: S5 File — (DOCX) [file pgph.0006694.s005.docx]

**Online** **Supplemental File 5.** Prevalence of chronic condition by age and sex groups (n = 5332).

| **Number of chronic conditions** | **% of Respondents by Number of Chronic conditions** | | | | | | | | | | ***N*** |
| --- | --- | --- | --- | --- | --- | --- | --- | --- | --- | --- | --- |
|  | **18-29 years** | | **30-44 years** | | **45-59 years** | | **≥ 60 years** | | **Total** | |  |
|  | **F** | **M** | **F** | **M** | **F** | **M** | **F** | **M** | **F** | **M** |  |
| 0 chronic condition | 44.6 | 78.6 | 42.3 | 54.2 | 33.8 | 27.5 | 16.3 | 9.7 | 40.3 | 56.5 | *2617* |
| 1 chronic condition | 50.8 | 19.6 | 41.6 | 33.9 | 41.3 | 45.3 | 34.9 | 40.8 | 45.0 | 29.8 | *1960* |
| 2 chronic conditions | 4.5 | 1.6 | 13.5 | 10.0 | 19.3 | 20.1 | 27.9 | 32.8 | 11.9 | 10.1 | *581* |
| 3+ chronic conditions | 0.1 | 0.2 | 2.6 | 1.8 | 5.6 | 7.1 | 20.9 | 16.8 | 2.8 | 3.6 | *174* |
| **≥ 2 chronic conditions**  **(multimorbidity)** | **4.6** | **1.8** | **16.1** | **11.8** | **24.9** | **27.2** | **48.8** | **49.6** | **14.7** | **13.7** | *755* |
| ***N*** | *960* | *1585* | *719* | *271* | *715* | *801* | *43* | *238* | *2437* | *2895* | *5332* |
| * F: Female; M: Male | | | | | | | | | | | |
