# Supplementary material for: Prevalence, patterns and associated behavioural risk factors of multimorbidity in rural India: Cross-sectional analysis from the Andhra Pradesh Children and Parents Study (APCAPS)
Source: PLOS Glob Public Health. 2026 Jul 30;6(7):e0006694. doi: 10.1371/journal.pgph.0006694 (PMC13422877; doi:10.1371/journal.pgph.0006694)
Supplement: S6 File — (DOCX) [file pgph.0006694.s006.docx]

**Online** **Supplemental File 6.** The distribution of BRFs between those with multimorbidity and those without multimorbidity (n = 5332).

| **Characteristics** | **With multimorbidity (n = 755) *n (%)*** | **Not with multimorbidity (n = 4557) *n (%)*** | ***Χ^2^ (P)*** |
| --- | --- | --- | --- |
| Daily drinking | | | |
| No | 730 (96.7) | 4492 (98.1) | 6.08  (0.01) |
| Yes | 25 (3.3) | 85 (1.9) |  |
| Tobacco consumption | | | |
| No | 416 (55.1) | 3532 (77.2) | 163.09  **(< 0.0001)** |
| Current or former | 339 (44.9) | 1045 (22.8) |  |
| Physical inactivity | | | |
| Active | 261 (34.6) | 1487 (32.5) | 1.18  (0.28) |
| Sedentary | 494 (65.4) | 3090 (67.5) |  |
| Poor sleep | | | |
| 6-10 hours / day | 687 (91.0) | 4325 (94.5) | 13.47  **(0.0002)** |
| <6 or ≥10 hours / day | 68 (9.0) | 252 (5.5) |  |
| * *Χ^2^*: Chi-squared | | | |
