# Supplementary material for: Prevalence, patterns and associated behavioural risk factors of multimorbidity in rural India: Cross-sectional analysis from the Andhra Pradesh Children and Parents Study (APCAPS)
Source: PLOS Glob Public Health. 2026 Jul 30;6(7):e0006694. doi: 10.1371/journal.pgph.0006694 (PMC13422877; doi:10.1371/journal.pgph.0006694)
Supplement: S7 File — (DOCX) [file pgph.0006694.s007.docx]

**Online** **Supplemental File 7.** Comparison between models of latent class analysis (LCA) (n = 5332).

| **N of Cluster** | **BIC** | **LL** | **df** | **npar** | **X^2** |
| --- | --- | --- | --- | --- | --- |
| **2** | **21995.90** | **-10882.1** | **5305** | **27** | **4868.29** |
| 3 | 22044.26 | -10846.06 | 5291 | 41 | 2580.70 |
| 4 | 22110.40 | -10820.07 | 5277 | 55 | 2265.23 |
| 5 | 22199.78 | -10824.66 | 5263 | 69 | 2355.38 |
| 6 | 22301.58 | -10785.60 | 5249 | 83 | 786.50 |
| 7 | 22392.44 | -10780.02 | 5235 | 97 | 704.13 |
| 8 | 22497.65 | -10774.22 | 5221 | 111 | 601.81 |
| 9 | 22617.79 | -10768.84 | 5207 | 125 | 987.06 |
| 10 | 22711.50 | -10756.36 | 5193 | 139 | 460.17 |
| * BIC: Bayesian information criterion; LL: Log-likelihood; df: Degree of freedom; npar: number of parameters | | | | | |
