# Supplementary material for: Prevalence, patterns and associated behavioural risk factors of multimorbidity in rural India: Cross-sectional analysis from the Andhra Pradesh Children and Parents Study (APCAPS)
Source: PLOS Glob Public Health. 2026 Jul 30;6(7):e0006694. doi: 10.1371/journal.pgph.0006694 (PMC13422877; doi:10.1371/journal.pgph.0006694)
Supplement: S8 File — (DOCX) [file pgph.0006694.s008.docx]

**Online** **Supplemental File 8.** The distribution of BRFs between those with the multimorbidity pattern and those without the multimorbidity pattern (n = 5332).

| **Behavioural risk factors (BRFs)** | **No anaemia or hypertension**  **(n = 3175) *n (%)*** | **Anaemia only**  **(n = 1096) *n (%)*** | **Hypertension only**  **(n = 841) *n (%)*** | **Pattern “Anaemia and Hypertension”**  **(n = 220) *n (%)*** | |
| --- | --- | --- | --- | --- | --- |
| Daily drinking | | | | |  |
| No (N = 5222) | 3152 (59.8) | 1089 (20.9) | 791 (15.1) | 219 (4.2) | |
| Yes (N = 110) | 52 (47.3) | 7 (6.4) | 50 (45.5) | 1 (0.9) | |
| Tobacco consumption | | | | |  |
| No (N = 3948) | 2405 (60.9) | 920 (23.3) | 487 (12.3) | 136 (3.4) | |
| Current or former (N = 1384) | 770 (55.6) | 176 (12.7) | 354 (25.6) | 84 (6.1) | |
| Physical inactivity | | | | |  |
| Active (N = 1748) | 1019 (58.3) | 371 (21.2) | 285 (16.3) | 73 (4.2) | |
| Sedentary (N = 3584) | 2156 (60.2) | 725 (20.2) | 556 (15.5) | 147 (4.1) | |
| Poor sleep | | | | |  |
| 6-10 hours / day (N = 5012) | 3010 (60.1) | 1007 (20.1) | 784 (15.6) | 211 (4.2) | |
| <6 or ≥10 hours / day (N = 320) | 165 (51.6) | 89 (27.8) | 57 (17.8) | 9 (2.8) | |
