# Supplementary material for: Prevalence, patterns and associated behavioural risk factors of multimorbidity in rural India: Cross-sectional analysis from the Andhra Pradesh Children and Parents Study (APCAPS)
Source: PLOS Glob Public Health. 2026 Jul 30;6(7):e0006694. doi: 10.1371/journal.pgph.0006694 (PMC13422877; doi:10.1371/journal.pgph.0006694)
Supplement: Attachment — Submitted filename: Response to Reviewers.docx [file pgph.0006694.s010.docx]

15/11/2025

Professor Nnodimele Onuigbo Atulomah

*PLOS Global Public Health*

Dear Professor Nnodimele Onuigbo Atulomah,

Thank you for facilitating the review of our manuscript entitled: ‘Behavioural risk factors for prevalence and patterns of multimorbidity in a rural population in India: Cross-sectional analysis from the Andhra Pradesh Children and Parents Study (APCAPS)’ which was submitted for consideration as an original research article in the *PLOS Global Public Health.*

We have read the editor and reviewers’ comments with great interest, and have revised our paper in light of these comments. We have addressed each comment, with full details of the revisions given below. These are also highlighted with the track change function in the revised manuscript (to which Line numbers refer).

**Editor(s)' Comments:**

- **Please ensure that your Ethics Statement is available in its entirety at the beginning of your Methods section, under a subheading 'Ethics Statement'. It must include:**
  1) **The name(s) of the Institutional Review Board(s) or Ethics Committee(s)**
  2) **The approval number(s), or a statement that approval was granted by the named board(s)**
  3) **(for human participants/donors) - A statement that formal consent was obtained (must state whether verbal/written) OR the reason consent was not obtained (e.g. anonymity).**
  - Thank you for this suggestion. We have added the “Ethics” sub-section in the Methods section in Lines 115-126, which now read: “Prior to data collection, approval had been sought from the village heads and their panchayats in each of the 29 villages. Written informed consent (or witnessed thumbprint if illiterate) was obtained from all participants. The APCAPS study received ethical approval from the National Institute of Nutrition (Hyderabad, India), the Indian Council of Medical Research (New Delhi, India), and the London School of Hygiene and Tropical Medicine (London, United Kingdom). This specific analysis was reviewed by the executive team of the Andhra Pradesh Children and Parents Study and the Ethics Board of the London School of Economics and Political Science (Approval Number No.44760). We accessed the data for research purposes on July 21, 2020”. We have amended the “Ethics approval statement” section of the manuscript in Lines 379-385, which now read: “The APCAPS study received ethical approval from the National Institute of Nutrition (Hyderabad, India), the Indian Council of Medical Research (New Delhi, India), and the London School of Economics and Political Science (London, United Kingdom). We accessed the data for research purposes on July 21, 2020. This study was reviewed by the executive team of the Andhra Pradesh Children and Parents Study and the Ethics Board of the London School of Economics and Political Science (Approval Number No. 44760). Formal consent was received from participants prior to data collection.”
- **2. We have noticed that you have uploaded Supporting Information files, but you have not included a list of legends. Please add a full list of legends for your Supporting Information files after the references list.**
  - Thank you for this suggestion. We have added a full list of legends for the supporting information files after the reference list in Lines 567-586.
- **For studies involving third-party data, we encourage authors to share any data specific to their analyses that they can legally distribute. PLOS recognizes, however, that authors may be using third-party data they do not have the rights to share. When third-party data cannot be publicly shared, authors must provide all information necessary for interested researchers to apply to gain access to the data (https://journals.plos.org/plosone/s/data-availability#loc-acceptable-data-access-restrictions). For any third-party data that the authors cannot legally distribute, they should include the following information in their Data Availability Statement upon submission:**
  1) **A description of the data set and the third-party source**
  2) **If applicable, verification of permission to use the data set**
  3) **Confirmation of whether the authors received any special privileges in accessing the data that other researchers would not have**
  4) **All necessary contact information others would need to apply to gain access to the data**
  - Thank you for this suggestion. We have amended the manuscript by providing all information necessary for interested researchers to apply to gain access to the data in Lines 379-381, which now read: “Permission to use the APCAPS data for this analysis was granted by the APCAPS Executive, with no special privileges in accessing the data that other researchers would not have. Data can be obtained by request from the executive team of the Andhra Pradesh Children and Parents Study: https://www.lshtm.ac.uk/research/centres-projects-groups/apcaps”.

**Reviewer 1:**

**The manuscript addresses a relevant area of public health and has the potential to make a stronger contribution with further refinement. In particular, more clarity in the methodology, a more developed introduction, and improvements in the general presentation would help strengthen the paper. My comments below are aimed at supporting the authors in this regard.**

- **Line 1-3: The title is somewhat ambiguous. Rephrasing it could make it easier to read and capture the essence of the study more effectively. For example: "Prevalence, Patterns and Associated Behavioural Risk Factors of Multimorbidity in Rural India: Insights from the Andhra Pradesh Children and Parents Study.”**
  - Thank you for this suggestion. We have amended the title as “Prevalence, Patterns and Associated Behavioural Risk Factors of Multimorbidity in Rural India: Insights from the Andhra Pradesh Children and Parents Study (APCAPS)”.
- **Line 39: The background context in the abstract is insufficient and could be expanded to better situate the study.**
  - Thank you for this suggestion. We have expanded the background context in the abstract in Lines 40-46, which now read: “The prevalence of multimorbidity is projected to increase substantially in India. Previous studies have focused on urban populations and older adults, who do not represent the majority of those at risk. We aimed to estimate the prevalence and investigate patterns of multimorbidity in a rural population in India and examine their associations with four common behavioural risk factors (BRFs): daily drinking, tobacco consumption, physical inactivity, and poor sleep.”
- **Line 43 - 49: The methods section of the abstract is clear on the data source, study design, and analysis. Adding details on the study population and main variables would make it more complete.**
  - Thank you for this suggestion. We have added details on the study population and main variables in the abstract in Lines 38-43, which now read: “We aimed to estimate the prevalence and investigate patterns of multimorbidity in a rural population in India and examine their associations with four common behavioural risk factors (BRFs): daily drinking, tobacco consumption, physical inactivity, and poor sleep. This cross-sectional analysis used data from all adult participants (age 18+ years) of the Andhra Pradesh Children and Parents Study (APCAPS) third follow-up wave, 2010-12”.
- **Line 49: The study population used in this secondary data analysis is not clear. APCAPS 2010-12 have the index children (18-21 years as of 2009-10), both parents and their siblings. Both parents and siblings were ∼6225 out of 10,213 participants. Please clearly define the adult population for which you observed a mean age of 35.5 years. Also, include the value for the standard deviation in the report of the age.**
  - Thank you for this suggestion. We have added the Flowchart of the analytical sample selection for the participants of the Andhra Pradesh Children and Parents Study (2010-2012) as the Online Supplemental File 1. We have added the value for the standard deviation in the report of the age in Lines 54-56, which now read: “Of 5332 adult participants (mean age 35.5 years, standard deviation 13.8 years) included in the analysis, 14.2% had multimorbidity, the prevalence of which increased steeply with age.” and in Lines 199-201, which now read: “The participants were 45.7% female and relatively young, with a median age of 31 years and a mean age of 35.5 years (interquartile range 23 to 47 years; standard deviation = 13.8)”. We have also added the plot for age distribution in Lines 201-202 and 553 (Online Supplemental File 4).
- **Line 54 - 60 and Line 216 - 225: Reporting only one pattern limits the study’s contribution, as multimorbidity research typically uncovers multiple clusters that can guide prevention and integrated care. Clarifying why only one pattern emerged from 13 conditions would strengthen the results and interpretation. This could also be briefly addressed in the discussion.**
  - Thank you for this insightful comment. We have expanded the Discussion section to clarify why only one pattern emerged. Compared to multimorbidity research that mostly focused on the older population, the study sample included in this analysis is relatively younger (mean age 35.5 years). This results in a lower prevalence of ageing-related chronic conditions, leading to a low prevalence of multimorbidity and to the formation of multiple clusters. We acknowledge that this low prevalence of most chronic conditions may potentially affect the pattern identification, and latent class analysis is mainly useful for identifing common clusters. We noted this as a limitation in the discussion section in Lines 334-342, which now read: “This may be due to the relatively young age of our study sample, which meant that ageing-related chronic conditions were less common. Also, this may result from the specific methodology we used to define a multimorbidity pattern, as some conditions, such as sarcopenia and diabetes, were represented but not prevalent in the multimorbidity cluster we identified. We note that the above comparisons between our findings and other literature on BRFs for multimorbidity should be interpreted with caution due to the wide variation in how different studies have defined multimorbidity, its specific patterns and how these are measured”.
- **Line 63 - 97: It comes across as though more attention was given to analysing the secondary data than to the application of theory related to risk behaviour or justifying the methodological approach for this study. Also, it would be helpful if the authors clarified early in the manuscript the distinction between multimorbidity (the presence of two or more chronic conditions in an individual) and multimorbidity patterns (the clustering of specific conditions across individuals). This would improve clarity for readers less familiar with the terminology.**
  - Thank you for this suggestion. We have amended the manuscript to strengthen the justification of our methodological approach and clarify the definitions used in the study. We highlighted that behavioural risk factors (BRFs) are “important targets in the prevention and management of multimorbidity, which is often comprised of chronic conditions that share common behavioural causes” in Lines 72-74. We detailed the use of latent class analysis, explaining that each latent class represents a particular pattern of multimorbidity in Lines 171-184, which now read: “To investigate multimorbidity patterns, we used Latent Class Analysis (LCA), an unsupervised machine learning approach for describing the relationships among categorical variables. LCA produces a probabilistic clustering of individuals into latent classes. Each latent class represents a particular pattern of chronic conditions. LCA simultaneously estimates the latent classes and the probability of class membership for every individual. Thus, LCA enabled us to identify conditions that were highly represented in the dataset and correlated with each other, from which we selected the most common conditions within each latent class to define multimorbidity patterns for subsequent regression analyses based on probabilistic statistics of each condition. A detailed explanation of the methodology is presented in Online Supplemental File 3. Participants without multimorbidity or those having no disease were included in LCA to minimise the overfitting of cluster analysis. Model selection was determined on the optimal Bayesian information criterion (BIC) values, with lower BIC values indicating a better-fitting model”. We have added the clarification of the distinction between multimorbidity and multimorbidity patterns in Lines 138-142, which now read: “ We defined multimorbidity as the co-existence of two or more chronic conditions in a single individual, which is the most common definition of multimorbidity used in epidemiological studies; and we define multimorbidity patterns as the specific probabilistic co-existence of chronic conditions across individuals.” and have expanded on why different BRFs might be linked to multimorbidity in the introduction in Lines 80-82 “Behavioural risk factors (BRFs) are likely to be important targets in the prevention and management of multimorbidity, which is often comprised of chronic conditions that share common behavioural causes”.
- **Line 91 - 92: The description of the study design is unclear. Since this is a secondary analysis of a longitudinal cohort, it would be clearer to state that you used a cross-sectional snapshot from the cohort data rather than cross-sectional data from the cohort. Also, the phrase “young and middle-aged adults” would be clearer if age ranges were provided.**
  - Thank you for this suggestion. We have improved the title and highlighted the manuscript by stating used a cross-sectional design from the cohort data and phrasing young and middle-aged adults in Lines 100-103, which now read: “We used cross-sectional data from the Andhra Pradesh Children and Parents Study (APCAPS), a rural population-based cohort including primarily young and middle-aged adults with detailed information on a range of BRFs, to examine the burden of multimorbidity and determine its BRFs in rural India”.
- **Line 105 - 111: Since this is a secondary data, the process of obtaining informed consent and ethical approval should be rephrased and the original source of information referenced appropriately.**
  - Thank you for this suggestion. We have improved the ethics approval statement in Lines 117-128, which now read: “Prior to data collection, approval had been sought from the village heads and their panchayats in each of the 29 villages. Written informed consent (or witnessed thumbprint if illiterate) was obtained from all participants. The APCAPS study received ethical approval from the National Institute of Nutrition (Hyderabad, India), the Indian Council of Medical Research (New Delhi, India), and the London School of Hygiene and Tropical Medicine (London, United Kingdom). This specific analysis was reviewed by the executive team of the Andhra Pradesh Children and Parents Study and the Ethics Board of the London School of Economics and Political Science (Approval Number No.44760). We accessed the data for research purposes on July 21, 2020.” and in Lines 405-411, which now read: “The APCAPS study received ethical approval from the National Institute of Nutrition (Hyderabad, India), the Indian Council of Medical Research (New Delhi, India), and the London School of Hygiene and Tropical Medicine (London, United Kingdom). We accessed the data for research purposes on July 21, 2020. This study was reviewed by the executive team of the Andhra Pradesh Children and Parents Study and the Ethics Board of the London School of Economics and Political Science (Approval Number No. 44760). Formal consent was received from participants prior to data collection.”.
- **Line 132 - 134: The text notes difficulties in measuring dietary qualities. It would be useful to specify what these difficulties were.**
  - Thank you for this suggestion. We have added the clarification in the methods section that diet was excluded due to specific difficulties in measuring and summarising dietary quality as a binary variable as consistent to other variables of behavioural risk factors in Lines 150-156, which now read: “We did not examine diet, another potential BRF for multimorbidity, because diet is highly complex and multifaceted and it was not deemed feasible to summarise dietary information into a meaningful binary indicator for dietary quality that could be analysed in a consistent manner alongside the other BRFs”. We noted this as further research in the discussion section in Lines 360-362: “We cannot rule out residual confounding, such as due to diet, genetic factors, or inaccurately measured socioeconomic factors, which could have inflated our observed associations” and in Lines 376-379: “Further research should take diet into analysis and aim to establish the causality of these associations to inform more targeted behavioural interventions to prevent and appropriately manage multimorbidity in this setting”.
- **Line 174: The analysis provides some useful insights, but the framing could be improved.**
  - Thank you for this suggestion. We have improved the overall presentation on the framing of the analysis, which reflected in the Results section (Lines 195-271).
- **Line 296 - 306: The discussion provides helpful context on why only one multimorbidity pattern emerged however, this could be strengthened by elaborating a little more on how the analytic choices may have influenced this outcome.**
  - Thank you for your suggestion. We have included the context in the Discussion sections in Lines 331-342: Our study did not find other multimorbidity patterns that have been recognised globally, such as “cardio-metabolic”, or specific patterns identified in India, such as “diabetes and hypertension” and “arthritis and hypertension”. This may be due to the relatively young age of our study sample, which meant that ageing-related chronic conditions were less common. Also, this may result from the specific methodology we used to define a multimorbidity pattern, as some conditions, such as sarcopenia and diabetes, were represented but not prevalent in the multimorbidity cluster we identified. We note that the above comparisons between our findings and other literature on BRFs for multimorbidity should be interpreted with caution due to the wide variation in how different studies have defined multimorbidity, its specific patterns and how these are measured”. We have amended the text on how the emerging multimorbidity pattern is influenced by the analytic choices in the Methods section in Line 176-179, which now read: “Thus, LCA enabled us to identify conditions that were highly represented in the dataset and correlated with each other, from which we selected the most common conditions within each latent class to define multimorbidity patterns for subsequent regression analyses based on probabilistic statistics of each condition”, with full methodology explanation in Online Supplemental File 3.

We believe that the modifications described above have strengthened the manuscript. We hope that our adjustments make the revised paper suitable for publication in the *PLOS Global Public Health* and we look forward to your decision.

Yours sincerely,

Mr Wenbo Song *On behalf of all co-authors*
