# Supplementary material for: Prevalence, patterns and associated behavioural risk factors of multimorbidity in rural India: Cross-sectional analysis from the Andhra Pradesh Children and Parents Study (APCAPS)
Source: PLOS Glob Public Health. 2026 Jul 30;6(7):e0006694. doi: 10.1371/journal.pgph.0006694 (PMC13422877; doi:10.1371/journal.pgph.0006694)
Supplement: Attachment — Submitted filename: Response to Reviewers 2.docx [file pgph.0006694.s011.docx]

09/03/2026

Dr Jennifer Tucker

*PLOS Global Public Health*

Dear Dr Jennifer Tucker,

Thank you for facilitating the review of our manuscript entitled: ‘Prevalence, Patterns and Associated Behavioural Risk Factors of Multimorbidity in Rural India: Cross-sectional analysis from the Andhra Pradesh Children and Parents Study (APCAPS)’, which was submitted for consideration as an original research article in the *PLOS Global Public Health.*

We have read the reviewers’ additional comments with great interest, and have further revised our paper in light of these comments. We have addressed each comment, with full details of the revisions given below. These are also highlighted with the track change function in the revised manuscript (to which Line numbers refer).

**Reviewers' Comments:**

**The authors have made several notable improvements in this revised manuscript. The title is now clearer and more informative, the abstract better outlines the study aims and findings, and the introduction, methods, and discussion are more transparent. Remaining areas for refinement are listed below:**

- **Lines 38-40: To better contextualise the study, the authors should add a brief phrase on the public health importance of multimorbidity, particularly in rural India.**
  - Thank you for this suggestion. We have expanded the introduction section on the public health importance of multimorbidity in rural India in Lines 68-72, which now reads: “The prevalence of multimorbidity in rural India is estimated to be as high as 54.8% in Punjab state (4), which is at more advanced stages of economic transition (ref). Multimorbidity has been identified as a substantial threat to fragile rural health infrastructures in India, potentially exacerbating health inequalities experienced by vulnerable households.”
- **Lines 44-52: Please revise “age 18+ years” to “aged ≥18 years” or include the age range to improve clarity and interpretation of the reported mean age of 35.5 years.**
  - Thank you for this suggestion. We have improved this in the abstract in Lines 41-43, which now reads: “This cross-sectional analysis used data of all adult participants (aged ≥18 years, mean age of 35.5 years) from the third follow-up of Andhra Pradesh Children and Parents Study (APCAPS), 2010-12.”, as well as have clarified this in the methods section in Line 116: “We included the data on all adult participants aged ≥18 years.”
- **Line 66-95: The introduction still leans more toward empirical motivation than theoretical framing. Defining multimorbidity patterns explicitly and including a theoretical framing alongside empirical motivation could improve clarity for readers less familiar with the terminology.**
  - Thank you for this suggestion. We have expanded the introduction section on defining multimorbidity patterns explicitly and including a theoretical framing alongside empirical motivation in Lines 93-99, which now reads: “Furthermore, despite evidence from global studies that specific patterns (combined clusters of chronic conditions) of multimorbidity may be associated with particular BRFs (7,8), this has not been investigated in the Indian context (14). This has hindered understanding of the causal links between specific clusters of conditions relevant to the Indian context, including their underlying BRFs, which may guide targets for interventions to prevent multimorbidity that are appropriate for this setting (15).”
- **Line 97-99: The description of the study design is still unclear. As previously stated, since APCAPS is a longitudinal cohort study, it would be more precise to describe this as a cross-sectional snapshot from the cohort data rather than cross-sectional data from the cohort. This clarifies that only one wave is used for the analysis and avoids potential misinterpretation of the study design.**
  - Thank you for this suggestion. We have clarified this in the introduction section in Lines 101-102: “We used cross-sectional data from the third wave of the Andhra Pradesh Children and Parents Study (APCAPS)” and in methods section in Lines 111-112 “The present cross-sectional analyses used the third wave data of the APCAPS cohort (see Online Supplemental File 1), a prospective study involving 29 rural towns and villages near Hyderabad city, Telangana state, India (15).” We have also amended the title as “Prevalence, Patterns and Associated Behavioural Risk Factors of Multimorbidity in Rural India: Cross-sectional analysis from the Andhra Pradesh Children and Parents Study (APCAPS)”.
- **Line 114- 124: The section clearly describes participant consent and ethical approvals for both the APCAPS cohort and this secondary analysis. To fully meet journal requirements, please cite the original APCAPS study/protocol where consent and approvals were obtained after mentioning it. Rename the subsection as “Ethics Statement”. Also, clarify that permission from village heads/panchayats is community approval, separate from formal ethics committee approval.**
  - Thank you for this suggestion. We have added the citation to the original APCAPS study in Line 113 and have renamed the subsection in Line 118 as “Ethics Statement”. We have separated the community approval from the committee approval in Lines 119-129, which now reads: “The APCAPS study received ethical approval from the National Institute of Nutrition (Hyderabad, India), the Indian Council of Medical Research (New Delhi, India), and the London School of Hygiene and Tropical Medicine (London, United Kingdom). Prior to data collection, community approval had been sought from the village heads and their panchayats in each of the 29 villages (15). Written informed consent (or witnessed thumbprint if illiterate) was obtained from all participants.” and “This analysis was reviewed by the executive team of the Andhra Pradesh Children and Parents Study and the Ethics Board of the London School of Economics and Political Science (Approval Number No.44760). We accessed the data for research purposes on July 21, 2020.”
- **Line 265-372: To strengthen the interpretation, it would be useful to briefly explain how the analytic choices, including model selection and included conditions, might have contributed to finding only one multimorbidity pattern.**
  - Thank you for this suggestion. We have added the explanation on how the analytic choices, including model selection and included conditions, might have contributed to finding only one multimorbidity pattern in Lines 353-359: “Although we used the most common clustering method, latent class analysis, for identifying multimorbidity patterns (45), the low prevalence of several chronic conditions limited the overall variance in co-occurrence of chronic conditions, resulting in a single multimorbidity pattern that primarily reflects disease burden rather than distinct phenotypic subtypes. The fact that we only included 13 conditions may have also limited our ability to detect certain patterns, especially those containing rarer diseases not captured in our study.”

We believe that the modifications described above have strengthened the manuscript. We hope that our adjustments make the revised paper suitable for publication in the *PLOS Global Public Health* and we look forward to your decision.

Yours sincerely,

Mr Wenbo Song *on behalf of all co-authors*
