# Supplementary material for: Prevalence, patterns and associated behavioural risk factors of multimorbidity in rural India: Cross-sectional analysis from the Andhra Pradesh Children and Parents Study (APCAPS)
Source: PLOS Glob Public Health. 2026 Jul 30;6(7):e0006694. doi: 10.1371/journal.pgph.0006694 (PMC13422877; doi:10.1371/journal.pgph.0006694)
Supplement: Attachment — Submitted filename: Response to Reviewers 3.docx [file pgph.0006694.s012.docx]

15/05/2026

Dr Helen Howard

*PLOS Global Public Health*

Dear Dr Helen Howard,

Thank you for facilitating the review of our manuscript entitled: ‘Prevalence, Patterns and Associated Behavioural Risk Factors of Multimorbidity in Rural India: Cross-sectional analysis from the Andhra Pradesh Children and Parents Study (APCAPS)’, which was submitted for consideration as an original research article in the *PLOS Global Public Health.*

We have read the reviewers’ additional comments with great interest, and have further revised our paper in light of these comments. We have addressed each comment, with full details of the revisions given below. These are also highlighted with the track change function in the revised manuscript.

**Reviewers' Comments:**

- **Reviewer #1: The authors have made significant revisions to this manuscript. I’m curious to know why the title was changed from: “Prevalence, Patterns and Associated Behavioural Risk Factors of Multimorbidity in Rural India: Insights from the Andhra Pradesh Children and Parents Study (APCAPS)” to “Prevalence, Patterns and Associated Behavioural Risk Factors of Multimorbidity in Rural India: Cross-sectional analysis from the Andhra Pradesh Children and Parents Study (APCAPS)”. Aside from that, I have no additional comments.**
  - Thank you for your comment. We changed the title to be more precise to clarify that only one follow-up wave is used for the analysis and to avoid potential misinterpretation of the study design.
- **Reviewer #2: The investigators have examined the data in a cross sectional way using the resources at hand. All the statistical elements are in place including the LCA which appears to have been accurately followed from supplemental file 3 and other multivariate (logistic) and multinomial approaches common to this type of investigation. Model selection by BIC is normal. The statistical approach was applied and executed properly given the restrictions of the sample. The sample from the 18 or older group may be somewhat restrictive and it would have been more informative knowing the underlying population number in this group. The final number of 5332 in Table 1 is certainly a small selection from the population. Also expanding the diagnoses or chronic conditions to more than just 13 would have maybe caught more morbidity patterns. Multimorbidity patterns may also become more evident taking a more longitudinal approach and examining approaches such as multimorbidity trajectories. The authors recognize this and it should be noted as a restriction. Also medication or adverse event associations could come into consideration.**
  - Thank you for your comment. We have included the information on data selection in Lines 194-197: “Of the 6,944 cohort participants recruited to APCAPS between 2010 and 2012, we excluded those under 18 years of age (n = 646) and those with missing values for any disease outcomes or BRFs (n = 966), resulting in 5332 participants included in the analyses (Table 1).” We have also explained our choices of these 13 conditions in Lines 132-133: “We examined 13 common chronic conditions, based on the availability of either or both self-reported history and symptom-based screening.” and in Lines 342-347: “Moreover, we included 13 common chronic conditions across all adult age groups, in comparison to earlier Indian studies in which the median number of included chronic conditions was 9 and focused on older populations (3). Furthermore, as many chronic conditions remain undiagnosed in India, we used validated clinical measures to classify 8 out of 13 chronic conditions, rather than relying solely on self-reported diagnoses as most previous studies have done (44).” We have emphasised the need for further longitudinal investigations in both the abstract and in Lines 348-350: “However, our data were cross-sectional, meaning that it is not possible to establish the temporal ordering of exposure and outcome; longitudinal studies are needed to clarify this.” We expanded the discussion section and added the point regarding medication or adverse event associations in Lines 353-354: “Existing medication uses or adverse event interactions can also bias associations by altering presentations of chronic conditions.”

We believe that the modifications described above have strengthened the manuscript. We hope that our adjustments make the revised paper suitable for publication in the *PLOS Global Public Health* and we look forward to your decision.

Yours sincerely,

Mr Wenbo Song *on behalf of all co-authors*
