# Supplementary material for: Prevalence, patterns and associated behavioural risk factors of multimorbidity in rural India: Cross-sectional analysis from the Andhra Pradesh Children and Parents Study (APCAPS)
Source: PLOS Glob Public Health. 2026 Jul 30;6(7):e0006694. doi: 10.1371/journal.pgph.0006694 (PMC13422877; doi:10.1371/journal.pgph.0006694)
Supplement: Peer review history

# Peer review: Decision Letter 0

**DOI:** 10.1371/journal.pgph.0006694.r001

**Authors:** Nnodimele Atulomah

2 Nov 2025

PGPH-D-25-01731

Behavioural risk factors for prevalence and patterns of multimorbidity in a rural population in India: Cross-sectional analysis from the Andhra Pradesh Children and Parents Study (APCAPS)

PLOS Global Public Health

Dear Mr. Song,

Thank you for submitting your manuscript to PLOS Global Public Health. After careful consideration, we feel that it has merit but does not fully meet PLOS Global Public Health’s publication criteria as it currently stands. Therefore, we invite you to submit a revised version of the manuscript that addresses the points raised during the review process.

Please submit your revised manuscript by 15th November 2025. If you will need more time than this to complete your revisions, please reply to this message or contact the journal office at globalpubhealth@plos.org. When you're ready to submit your revision, log on to https://www.editorialmanager.com/pgph/ and select the 'Submissions Needing Revision' folder to locate your manuscript file.

Please include the following items when submitting your revised manuscript:

- A rebuttal letter that responds to each point raised by the editor and reviewer(s). You should upload this letter as a separate file labeled 'Response to Reviewers'.
- A marked-up copy of your manuscript that highlights changes made to the original version. You should upload this as a separate file labeled 'Revised Manuscript with Track Changes'.
- An unmarked version of your revised paper without tracked changes. You should upload this as a separate file labeled 'Manuscript'.

Guidelines for resubmitting your figure files are available below the reviewer comments at the end of this letter.

We look forward to receiving your revised manuscript.

Kind regards,

Nnodimele Onuigbo Atulomah, PhD

Academic Editor

PLOS Global Public Health

Journal Requirements:

1. Please ensure that your Ethics Statement is available in its entirety at the beginning of your Methods section, under a subheading 'Ethics Statement'. It must include:

1) The name(s) of the Institutional Review Board(s) or Ethics Committee(s)

2) The approval number(s), or a statement that approval was granted by the named board(s)

3) (for human participants/donors) - A statement that formal consent was obtained (must state whether verbal/written) OR the reason consent was not obtained (e.g. anonymity).

2. We have noticed that you have uploaded Supporting Information files, but you have not included a list of legends. Please add a full list of legends for your Supporting Information files after the references list.

3. For studies involving third-party data, we encourage authors to share any data specific to their analyses that they can legally distribute. PLOS recognizes, however, that authors may be using third-party data they do not have the rights to share. When third-party data cannot be publicly shared, authors must provide all information necessary for interested researchers to apply to gain access to the data. (https://journals.plos.org/plosone/s/data-availability#loc-acceptable-data-access-restrictions)

For any third-party data that the authors cannot legally distribute, they should include the following information in their Data Availability Statement upon submission:

1) A description of the data set and the third-party source

2) If applicable, verification of permission to use the data set

3) Confirmation of whether the authors received any special privileges in accessing the data that other researchers would not have

4) All necessary contact information others would need to apply to gain access to the data

If the reviewer comments include a recommendation to cite specific previously published works, please review and evaluate these publications to determine whether they are relevant and should be cited. There is no requirement to cite these works unless the editor has indicated otherwise.

Additional Editor Comments (if provided):

Kindly study the reviewer's observations and comments and revise the submision. Unfortunately, the manuscript lacked any theoretical considerations to explain the behavioural risk factors which has limited the needed contributions it could have made. From an epidemiological point of view, behavioural risk factors, more of modifiable risk for multimorbidity has been raised, it would have been appropriate to provide scientific-based insight regarding this in terms of the dynamics involved and not merely mentioning the behaviour alone. What are the antecedent sources of the behavoural risk factors? The current state of the manuscript seriously lack sound theoretical framework adequate to elucidate the dynamics and rationale for the behavioural risk factors in a scientific way.

[Note: HTML markup is below. Please do not edit.]

Reviewers' comments:

Reviewer's Responses to Questions

**Comments to the Author**

1. Does this manuscript meet PLOS Global Public Health’s publication criteria? Is the manuscript technically sound, and do the data support the conclusions? The manuscript must describe methodologically and ethically rigorous research with conclusions that are appropriately drawn based on the data presented.

Reviewer #1: No

**********

2. Has the statistical analysis been performed appropriately and rigorously?

Reviewer #1: I don't know

**********

3. Have the authors made all data underlying the findings in their manuscript fully available (please refer to the Data Availability Statement at the start of the manuscript PDF file)?

The PLOS Data policy requires authors to make all data underlying the findings described in their manuscript fully available without restriction, with rare exception. The data should be provided as part of the manuscript or its supporting information, or deposited to a public repository. For example, in addition to summary statistics, the data points behind means, medians and variance measures should be available. If there are restrictions on publicly sharing data—e.g. participant privacy or use of data from a third party—those must be specified.

Reviewer #1: No

**********

4. Is the manuscript presented in an intelligible fashion and written in standard English?

PLOS Global Public Health does not copyedit accepted manuscripts, so the language in submitted articles must be clear, correct, and unambiguous. Any typographical or grammatical errors should be corrected at revision, so please note any specific errors here.

Reviewer #1: No

**********

5. Review Comments to the Author

Please use the space provided to explain your answers to the questions above. You may also include additional comments for the author, including concerns about dual publication, research ethics, or publication ethics. (Please upload your review as an attachment if it exceeds 20,000 characters)

Reviewer #1: The manuscript addresses a relevant area of public health and has the potential to make a stronger contribution with further refinement. In particular, more clarity in the methodology, a more developed introduction, and improvements in the general presentation would help strengthen the paper. My comments below are aimed at supporting the authors in this regard.

Line 1-3: The title is somewhat ambiguous. Rephrasing it could make it easier to read and capture the essence of the study more effectively. For example: "Prevalence, Patterns and Associated Behavioural Risk Factors of Multimorbidity in Rural India: Insights from the Andhra Pradesh Children and Parents Study.”

Line 39: The background context in the abstract is insufficient and could be expanded to better situate the study.

Line 43 - 49: The methods section of the abstract is clear on the data source, study design, and analysis. Adding details on the study population and main variables would make it more complete.

Line 49: The study population used in this secondary data analysis is not clear. APCAPS 2010-12 have the index children (18-21 years as of 2009-10), both parents and their siblings. Both parents and siblings were ∼6225 out of 10,213 participants. Please clearly define the adult population for which you observed a mean age of 35.5 years. Also, include the value for the standard deviation in the report of the age.

Line 54 - 60 and Line 216 - 225: Reporting only one pattern limits the study’s contribution, as multimorbidity research typically uncovers multiple clusters that can guide prevention and integrated care. Clarifying why only one pattern emerged from 13 conditions would strengthen the results and interpretation. This could also be briefly addressed in the discussion.

Line 63 - 97: It comes across as though more attention was given to analysing the secondary data than to the application of theory related to risk behaviour or justifying the methodological approach for this study. Also, it would be helpful if the authors clarified early in the manuscript the distinction between multimorbidity (the presence of two or more chronic conditions in an individual) and multimorbidity patterns (the clustering of specific conditions across individuals). This would improve clarity for readers less familiar with the terminology.

Line 91 - 92: The description of the study design is unclear. Since this is a secondary analysis of a longitudinal cohort, it would be clearer to state that you used a cross-sectional snapshot from the cohort data rather than cross-sectional data from the cohort. Also, the phrase “young and middle-aged adults” would be clearer if age ranges were provided.

Line 105 - 111: Since this is a secondary data, the process of obtaining informed consent and ethical approval should be rephrased and the original source of information referenced appropriately.

Line 132 - 134: The text notes difficulties in measuring dietary qualities. It would be useful to specify what these difficulties were.

Line 174: The analysis provides some useful insights, but the framing could be improved.

Line 296 - 306: The discussion provides helpful context on why only one multimorbidity pattern emerged however, this could be strengthened by elaborating a little more on how the analytic choices may have influenced this outcome.

**********

6. PLOS authors have the option to publish the peer review history of their article (what does this mean?). If published, this will include your full peer review and any attached files.

**Do you want your identity to be public for this peer review?**  If you choose “no”, your identity will remain anonymous but your review may still be made public.

For information about this choice, including consent withdrawal, please see our Privacy Policy.

Reviewer #1: No

**********

[NOTE: If reviewer comments were submitted as an attachment file, they will be attached to this email and accessible via the submission site. Please log into your account, locate the manuscript record, and check for the action link "View Attachments". If this link does not appear, there are no attachment files.]

-->Figure Resubmissions:

While revising your submission, we strongly recommend that you use PLOS’s NAAS tool (https://ngplosjournals.pagemajik.ai/artanalysis) to test your figure files. NAAS can convert your figure files to the TIFF file type and meet basic requirements (such as print size, resolution), or provide you with a report on issues that do not meet our requirements and that NAAS cannot fix.

After uploading your figures to PLOS’s NAAS tool - https://ngplosjournals.pagemajik.ai/artanalysis, NAAS will process the files provided and display the results in the "Uploaded Files" section of the page as the processing is complete. If the uploaded figures meet our requirements (or NAAS is able to fix the files to meet our requirements), the figure will be marked as "fixed" above. If NAAS is unable to fix the files, a red "failed" label will appear above. When NAAS has confirmed that the figure files meet our requirements, please download the file via the download option, and include these NAAS processed figure files when submitting your revised manuscript.

# Author comment: Author response to Decision Letter 1

**DOI:** 10.1371/journal.pgph.0006694.r002

21 Dec 2025

Attachment Submitted filename: Response to Reviewers.docx

# Peer review: Decision Letter 1

**DOI:** 10.1371/journal.pgph.0006694.r003

**Authors:** Nnodimele Atulomah, Jennifer Tucker

3 Mar 2026

PGPH-D-25-01731R1

Prevalence, Patterns and Associated Behavioural Risk Factors of Multimorbidity in Rural India: Insights from the Andhra Pradesh Children and Parents Study (APCAPS)

PLOS Global Public Health

Dear Dr. Song,

Thank you for submitting your manuscript to PLOS Global Public Health. After careful consideration, we feel that it has merit but does not fully meet PLOS Global Public Health’s publication criteria as it currently stands. Therefore, we invite you to submit a revised version of the manuscript that addresses the points raised during the review process.

Please address the reviewers additional comments following review of your revised manuscript. They recommend additional revisions to provide clearer theoretical framing of the study for readers, to clearly specify the study design, and to further explain the analytical choices including model selection and the impact of this on the results.

Please submit your revised manuscript by Apr 01 2026 11:59PM. If you will need more time than this to complete your revisions, please reply to this message or contact the journal office at globalpubhealth@plos.org. When you're ready to submit your revision, log on to https://www.editorialmanager.com/pgph/ and select the 'Submissions Needing Revision' folder to locate your manuscript file.

Please include the following items when submitting your revised manuscript:

- A letter that responds to each point raised by the editor and reviewer(s). You should upload this letter as a separate file labeled 'Response to Reviewers'.
- A marked-up copy of your manuscript that highlights changes made to the original version. You should upload this as a separate file labeled 'Revised Manuscript with Track Changes'.
- An unmarked version of your revised paper without tracked changes. You should upload this as a separate file labeled 'Manuscript'.

Guidelines for resubmitting your figure files are available below the reviewer comments at the end of this letter.

We look forward to receiving your revised manuscript.

Kind regards,

Jennifer Tucker, PhD

Staff Editor

PLOS Global Public Health

Journal Requirements:

If the reviewer comments include a recommendation to cite specific previously published works, please review and evaluate these publications to determine whether they are relevant and should be cited. There is no requirement to cite these works unless the editor has indicated otherwise.

Please review your reference list to ensure that it is complete and correct. If you have cited papers that have been retracted, please include the rationale for doing so in the manuscript text, or remove these references and replace them with relevant current references. Any changes to the reference list should be mentioned in the rebuttal letter that accompanies your revised manuscript. If you need to cite a retracted article, indicate the article’s retracted status in the References list and also include a citation and full reference for the retraction notice.

Additional Editor Comments (if provided):

[Note: HTML markup is below. Please do not edit.]

Reviewers' comments:

Reviewer's Responses to Questions

**Comments to the Author**

1. If the authors have adequately addressed your comments raised in a previous round of review and you feel that this manuscript is now acceptable for publication, you may indicate that here to bypass the “Comments to the Author” section, enter your conflict of interest statement in the “Confidential to Editor” section, and submit your "Accept" recommendation.

Reviewer #1: (No Response)

**********

2. Does this manuscript meet PLOS Global Public Health’s publication criteria? Is the manuscript technically sound, and do the data support the conclusions? The manuscript must describe methodologically and ethically rigorous research with conclusions that are appropriately drawn based on the data presented.

Reviewer #1: Yes

**********

3. Has the statistical analysis been performed appropriately and rigorously?

Reviewer #1: (No Response)

**********

4. Have the authors made all data underlying the findings in their manuscript fully available (please refer to the Data Availability Statement at the start of the manuscript PDF file)?

The PLOS Data policy requires authors to make all data underlying the findings described in their manuscript fully available without restriction, with rare exception. The data should be provided as part of the manuscript or its supporting information, or deposited to a public repository. For example, in addition to summary statistics, the data points behind means, medians and variance measures should be available. If there are restrictions on publicly sharing data—e.g. participant privacy or use of data from a third party—those must be specified.

Reviewer #1: Yes

**********

5. Is the manuscript presented in an intelligible fashion and written in standard English?

PLOS Global Public Health does not copyedit accepted manuscripts, so the language in submitted articles must be clear, correct, and unambiguous. Any typographical or grammatical errors should be corrected at revision, so please note any specific errors here.

Reviewer #1: Yes

**********

6. Review Comments to the Author

Please use the space provided to explain your answers to the questions above. You may also include additional comments for the author, including concerns about dual publication, research ethics, or publication ethics. (Please upload your review as an attachment if it exceeds 20,000 characters)

Reviewer #1: The authors have made several notable improvements in this revised manuscript. The title is now clearer and more informative, the abstract better outlines the study aims and findings, and the introduction, methods, and discussion are more transparent.

Remaining areas for refinement are listed below:

Lines 38-40: To better contextualise the study, the authors should add a brief phrase on the public health importance of multimorbidity, particularly in rural India.

Lines 44-52: Please revise “age 18+ years” to “aged ≥18 years” or include the age range to improve clarity and interpretation of the reported mean age of 35.5 years.

Line 66-95: The introduction still leans more toward empirical motivation than theoretical framing. Defining multimorbidity patterns explicitly and including a theoretical framing alongside empirical motivation could improve clarity for readers less familiar with the terminology.

Line 97-99: The description of the study design is still unclear. As previously stated, since APCAPS is a longitudinal cohort study, it would be more precise to describe this as a cross-sectional snapshot from the cohort data rather than cross-sectional data from the cohort. This clarifies that only one wave is used for the analysis and avoids potential misinterpretation of the study design.

Line 114- 124: The section clearly describes participant consent and ethical approvals for both the APCAPS cohort and this secondary analysis. To fully meet journal requirements, please cite the original APCAPS study/protocol where consent and approvals were obtained after mentioning it. Rename the subsection as “Ethics Statement”. Also, clarify that permission from village heads/panchayats is community approval, separate from formal ethics committee approval.

Line 265-372: To strengthen the interpretation, it would be useful to briefly explain how the analytic choices, including model selection and included conditions, might have contributed to finding only one multimorbidity pattern.

**********

7. PLOS authors have the option to publish the peer review history of their article (what does this mean?). If published, this will include your full peer review and any attached files.

**Do you want your identity to be public for this peer review?**  If you choose “no”, your identity will remain anonymous but your review may still be made public.

For information about this choice, including consent withdrawal, please see our Privacy Policy.

Reviewer #1: No

**********

[NOTE: If reviewer comments were submitted as an attachment file, they will be attached to this email and accessible via the submission site. Please log into your account, locate the manuscript record, and check for the action link "View Attachments". If this link does not appear, there are no attachment files.]

Figure Resubmissions:

While revising your submission, we strongly recommend that you use PLOS’s NAAS tool (https://ngplosjournals.pagemajik.ai/artanalysis) to test your figure files. NAAS can convert your figure files to the TIFF file type and meet basic requirements (such as print size, resolution), or provide you with a report on issues that do not meet our requirements and that NAAS cannot fix.

After uploading your figures to PLOS’s NAAS tool - https://ngplosjournals.pagemajik.ai/artanalysis, NAAS will process the files provided and display the results in the "Uploaded Files" section of the page as the processing is complete. If the uploaded figures meet our requirements (or NAAS is able to fix the files to meet our requirements), the figure will be marked as "fixed" above. If NAAS is unable to fix the files, a red "failed" label will appear above. When NAAS has confirmed that the figure files meet our requirements, please download the file via the download option, and include these NAAS processed figure files when submitting your revised manuscript.

# Author comment: Author response to Decision Letter 2

**DOI:** 10.1371/journal.pgph.0006694.r004

22 Mar 2026

Attachment Submitted filename: Response to Reviewers 2.docx

# Peer review: Decision Letter 2

**DOI:** 10.1371/journal.pgph.0006694.r005

**Authors:** Nnodimele Atulomah, Jennifer Tucker, Helen Howard

26 Apr 2026

PGPH-D-25-01731R2

Prevalence, Patterns and Associated Behavioural Risk Factors of Multimorbidity in Rural India: Cross-sectional analysis from the Andhra Pradesh Children and Parents Study (APCAPS)

PLOS Global Public Health

Dear Dr. Song,

Thank you for submitting your manuscript to PLOS Global Public Health. After careful consideration, we feel that it has merit but does not fully meet PLOS Global Public Health’s publication criteria as it currently stands. Therefore, we invite you to submit a revised version of the manuscript that addresses the points raised during the review process.

Please submit your revised manuscript by May 24 2026 11:59PM. If you will need more time than this to complete your revisions, please reply to this message or contact the journal office at globalpubhealth@plos.org. When you're ready to submit your revision, log on to https://www.editorialmanager.com/pgph/ and select the 'Submissions Needing Revision' folder to locate your manuscript file.

Please include the following items when submitting your revised manuscript:

- A letter that responds to each point raised by the editor and reviewer(s). You should upload this letter as a separate file labeled 'Response to Reviewers'.
- A marked-up copy of your manuscript that highlights changes made to the original version. You should upload this as a separate file labeled 'Revised Manuscript with Track Changes'.
- An unmarked version of your revised paper without tracked changes. You should upload this as a separate file labeled 'Manuscript'.

Guidelines for resubmitting your figure files are available below the reviewer comments at the end of this letter.

As the corresponding author, your ORCID iD is verified in the submission system and will appear in the published article. PLOS supports the use of ORCID, and we encourage all coauthors to register for an ORCID iD and use it as well. Please encourage your coauthors to verify their ORCID iD within the submission system before final acceptance, as unverified ORCID iDs will not appear in the published article. *Only*  the individual author can complete the verification step; PLOS staff *cannot*  verify ORCID iDs on behalf of authors.

We look forward to receiving your revised manuscript.

Kind regards,

Helen Howard

Staff Editor

PLOS Global Public Health

Journal Requirements:

If the reviewer comments include a recommendation to cite specific previously published works, please review and evaluate these publications to determine whether they are relevant and should be cited. There is no requirement to cite these works unless the editor has indicated otherwise.

Please review your reference list to ensure that it is complete and correct. If you have cited papers that have been retracted, please include the rationale for doing so in the manuscript text, or remove these references and replace them with relevant current references. Any changes to the reference list should be mentioned in the rebuttal letter that accompanies your revised manuscript. If you need to cite a retracted article, indicate the article’s retracted status in the References list and also include a citation and full reference for the retraction notice.

Additional Editor Comments (if provided):

[Note: HTML markup is below. Please do not edit.]

Reviewers' comments:

Reviewer's Responses to Questions

**Comments to the Author**

1. If the authors have adequately addressed your comments raised in a previous round of review and you feel that this manuscript is now acceptable for publication, you may indicate that here to bypass the “Comments to the Author” section, enter your conflict of interest statement in the “Confidential to Editor” section, and submit your "Accept" recommendation.

Reviewer #1: All comments have been addressed

Reviewer #2: All comments have been addressed

**********

2. Does this manuscript meet PLOS Global Public Health’s publication criteria? Is the manuscript technically sound, and do the data support the conclusions? The manuscript must describe methodologically and ethically rigorous research with conclusions that are appropriately drawn based on the data presented.

Reviewer #1: Yes

Reviewer #2: Yes

**********

3. Has the statistical analysis been performed appropriately and rigorously?

Reviewer #1: (No Response)

Reviewer #2: Yes

**********

4. Have the authors made all data underlying the findings in their manuscript fully available (please refer to the Data Availability Statement at the start of the manuscript PDF file)?

The PLOS Data policy requires authors to make all data underlying the findings described in their manuscript fully available without restriction, with rare exception. The data should be provided as part of the manuscript or its supporting information, or deposited to a public repository. For example, in addition to summary statistics, the data points behind means, medians and variance measures should be available. If there are restrictions on publicly sharing data—e.g. participant privacy or use of data from a third party—those must be specified.

Reviewer #1: Yes

Reviewer #2: Yes

**********

5. Is the manuscript presented in an intelligible fashion and written in standard English?

PLOS Global Public Health does not copyedit accepted manuscripts, so the language in submitted articles must be clear, correct, and unambiguous. Any typographical or grammatical errors should be corrected at revision, so please note any specific errors here.

Reviewer #1: Yes

Reviewer #2: Yes

**********

6. Review Comments to the Author

Please use the space provided to explain your answers to the questions above. You may also include additional comments for the author, including concerns about dual publication, research ethics, or publication ethics. (Please upload your review as an attachment if it exceeds 20,000 characters)

Reviewer #1: The authors have made significant revisions to this manuscript. I’m curious to know why the title was changed from: “Prevalence, Patterns and Associated Behavioural Risk Factors of Multimorbidity in Rural India: Insights from the Andhra Pradesh Children and Parents Study (APCAPS)” to “Prevalence, Patterns and Associated Behavioural Risk Factors of Multimorbidity in Rural India: Cross-sectional analysis from the Andhra Pradesh Children and Parents Study (APCAPS)”. Aside from that, I have no additional comments.

Reviewer #2: The investigators have examined the data in a cross sectional way using the resources at hand. All the statistical elements are in place including the LCA which appears to have been accurately followed from supplemental file 3 and other multivariate (logistic) and multinomial approaches common to this type of investigation. Model selection by BIC is normal. The statistical approach was applied and executed properly given the restrictions of the sample.

The sample from the 18 or older group may be somewhat restrictive and it would have been more informative knowing the underlying population number in this group. The final number of 5332 in Table 1 is certainly a small selection from the population. Also expanding the diagnoses or chronic conditions to more than just 13 would have maybe caught more morbidity patterns. Multimorbidity patterns may also become more evident taking a more longitudinal approach and examining approaches such as multimorbidity trajectories. The authors recognize this and it should be noted as a restriction. Also medication or adverse event associations could come into consideration.

**********

7. PLOS authors have the option to publish the peer review history of their article (what does this mean?). If published, this will include your full peer review and any attached files.

**Do you want your identity to be public for this peer review?**  If you choose “no”, your identity will remain anonymous but your review may still be made public.

For information about this choice, including consent withdrawal, please see our Privacy Policy.

Reviewer #1: No

Reviewer #2: No

**********

[NOTE: If reviewer comments were submitted as an attachment file, they will be attached to this email and accessible via the submission site. Please log into your account, locate the manuscript record, and check for the action link "View Attachments". If this link does not appear, there are no attachment files.]

Figure Resubmissions:

While revising your submission, we strongly recommend that you use PLOS’s NAAS tool (https://ngplosjournals.pagemajik.ai/artanalysis) to test your figure files. NAAS can convert your figure files to the TIFF file type and meet basic requirements (such as print size, resolution), or provide you with a report on issues that do not meet our requirements and that NAAS cannot fix.

After uploading your figures to PLOS’s NAAS tool - https://ngplosjournals.pagemajik.ai/artanalysis, NAAS will process the files provided and display the results in the "Uploaded Files" section of the page as the processing is complete. If the uploaded figures meet our requirements (or NAAS is able to fix the files to meet our requirements), the figure will be marked as "fixed" above. If NAAS is unable to fix the files, a red "failed" label will appear above. When NAAS has confirmed that the figure files meet our requirements, please download the file via the download option, and include these NAAS processed figure files when submitting your revised manuscript.

# Author comment: Author response to Decision Letter 3

**DOI:** 10.1371/journal.pgph.0006694.r006

23 May 2026

Attachment Submitted filename: Response to Reviewers 3.docx

# Editor report: Decision Letter 3

**DOI:** 10.1371/journal.pgph.0006694.r007

**Authors:** Nnodimele Atulomah, Jennifer Tucker, Helen Howard, Julia Robinson

2 Jun 2026

Prevalence, Patterns and Associated Behavioural Risk Factors of Multimorbidity in Rural India: Cross-sectional analysis from the Andhra Pradesh Children and Parents Study (APCAPS)

PGPH-D-25-01731R3

Dear Mr Song,

We are pleased to inform you that your manuscript 'Prevalence, Patterns and Associated Behavioural Risk Factors of Multimorbidity in Rural India: Cross-sectional analysis from the Andhra Pradesh Children and Parents Study (APCAPS)' has been provisionally accepted for publication in PLOS Global Public Health.

Before your manuscript can be formally accepted you will need to complete some formatting changes, which you will receive in a follow up email. A member of our team will be in touch with a set of requests.

Please note that your manuscript will not be scheduled for publication until you have made the required changes, so a swift response is appreciated.

IMPORTANT: The editorial review process is now complete. PLOS will only permit corrections to spelling, formatting or significant scientific errors from this point onwards. Requests for major changes, or any which affect the scientific understanding of your work, will cause delays to the publication date of your manuscript.

If your institution or institutions have a press office, please notify them about your upcoming paper to help maximize its impact. If they'll be preparing press materials, please inform our press team as soon as possible -- no later than 48 hours after receiving the formal acceptance. Your manuscript will remain under strict press embargo until 2 pm Eastern Time on the date of publication. For more information, please contact globalpubhealth@plos.org.

Thank you again for supporting Open Access publishing; we are looking forward to publishing your work in PLOS Global Public Health.

Best regards,

Julia Robinson

Executive Editor

PLOS Global Public Health

***********************************************************

Reviewer Comments (if any, and for reference):
